# Contour Propagation Using Feature-Based Deformable Registration for Lung Cancer

**DOI:** 10.1155/2013/701514

**Published:** 2013-12-02

**Authors:** Yuhan Yang, Shoujun Zhou, Peng Shang, En Qi, Shibin Wu, Yaoqin Xie

**Affiliations:** Key Laboratory for Health Informatics, Shenzhen Institutes of Advanced Technology, Chinese Academy of Sciences, Shenzhen 518055, China

## Abstract

Accurate target delineation of CT image is a critical step in radiotherapy treatment planning. This paper describes a novel strategy for automatic contour propagation, based on deformable registration, for CT images of lung cancer. The proposed strategy starts with a manual-delineated contour in one slice of a 3D CT image. By means of feature-based deformable registration, the initial contour in other slices of the image can be propagated automatically, and then refined by active contour approach. Three algorithms are employed in the strategy: the Speeded-Up Robust Features (SURF), Thin-Plate Spline (TPS), and an adapted active contour (Snake), used to refine and modify the initial contours. Five pulmonary cancer cases with about 400 slices and 1000 contours have been used to verify the proposed strategy. Experiments demonstrate that the proposed strategy can improve the segmentation performance in the pulmonary CT images. Jaccard similarity (JS) mean is about 0.88 and the maximum of Hausdorff distance (HD) is about 90%. In addition, delineation time has been considerably reduced. The proposed feature-based deformable registration method in the automatic contour propagation improves the delineation efficiency significantly.

## 1. Introduction

Carcinoma of the lung is one of the most common cancers, which has the highest mortality rate all over the world [1]. Radiotherapy is an effective option for carcinoma treatment. How to maintain adequate sparing of the sensitive structures is one of the biggest challenges in radiotherapy, which can be faced by means of treatment planning [2, 3]. The precise target delineation is an essential prerequisite for treatment planning; this, in fact, provides dose escalation to the tumor [4]. In the conventional radiotherapy planning, the clinical contour delineation is manually conducted by physicians slice by slice; however, this is a tedious and time-consuming procedure. Consequently, some fully automatic methods on each slice, such as the traditional water-based segmentation and level set active contour, have been proposed. These techniques mainly rely on local image features, such as intensity and gradient variations. However, these methods are unable to generate accurate target contour in absence of distinctive local image feature, such as in the tissues of mediastinum, hilus pulmonis, or pulmonary artery.

For target delineation of metastatic lung cancer, the hilus pulmonis should be embraced in the lung. Some statistical model-based approaches, such as the classical active shape models [5] or active appearance models [6], have been proposed by adding superior contour constraints in procrustes analysis and principal component analysis. However, lung morphology varies from patient to patient; several efforts have been made to solve this issue by providing prior knowledge by means of three-dimensional views [7–10]. For example, Brown et al. [7] presented an automatic knowledge-based method for segmenting chest CT datasets. Then, Zhang et al. [8] used an anatomic pulmonary atlas, encoded with a priori information on the pulmonary anatomy, to automatically segment the oblique lobar fissures. Qazi et al. [9] presented a fully automatic hybrid approach and combined deformable registration with the model-based approach to accurately segment normal tissues and target from head and neck CT images. Additionally, Collins et al. [10] presented a 3D model-based segmentation method for the automatic identification and delineation of gross anatomical structures of the human brain. However, since these methods partially depended on a massive patient model dataset and complicated statistical analysis, the necessary work tends to be extensive, and it may result in potential errors arising from the reliability of the selection of the optimal model. In addition, three-dimensional image processing is a time-consuming procedure, and its accuracy hardly reaches the two-dimensional processing accuracy because of the deformation due to respiratory movement. As any error at this step is systematic and would affect the whole course of radiotherapy, manual delineation method is required.

In this paper, a 2D contour propagation method is proposed. Feature-based deformable registration method was employed by using an initial manual delineated contour slice as the prior knowledge; in this way the interaction time compared to the fully manual delineation method can be greatly reduced.

## 2. Materials and Methods

### 2.1. Overview of the Automatic Target Delineation

**Figure 1:**
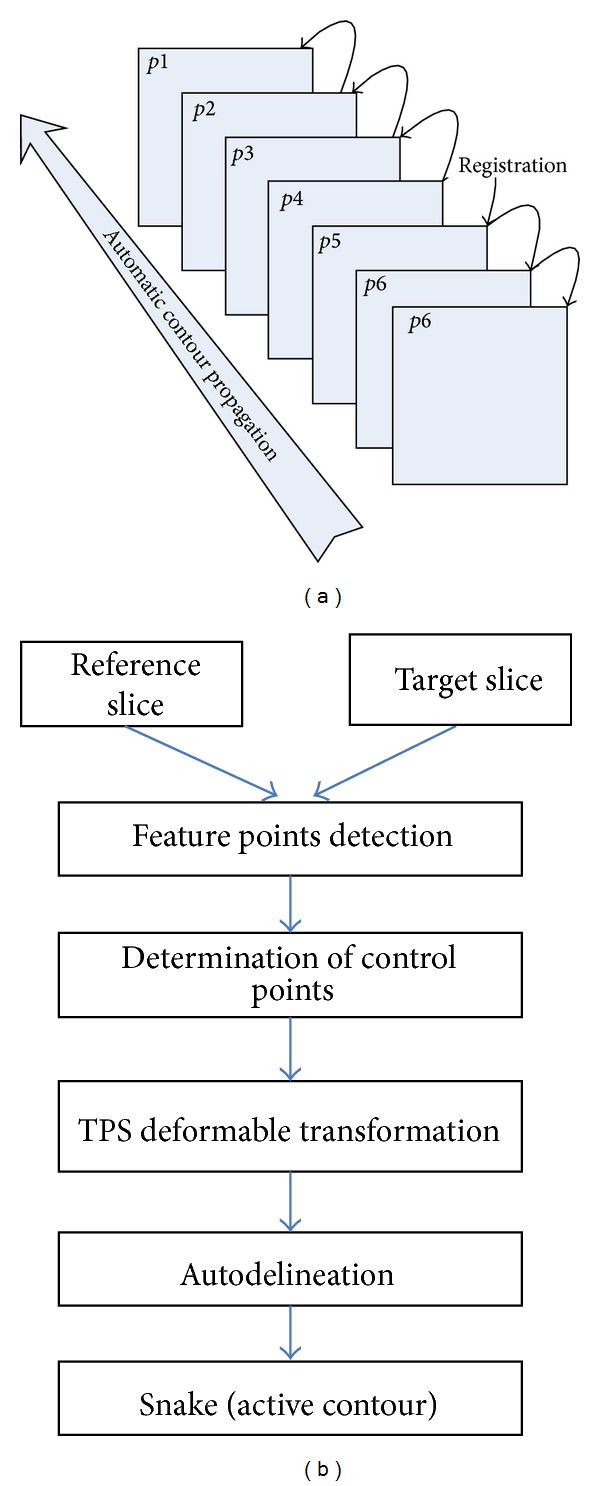
Flowchart of the automatic contour propagation method. (a) General flowchart of the automatic contour propagation; (b) flowchart of the automatic contour delineation between adjacent slices on (a).

The flowchart of the proposed automatic contour propagation is shown in Figure 1(a). In this study, a template-based registration method was introduced from the research of brain functional area [11], in which a standard brain is used as template to determine the brain functional area for clinical cases through deformable registration. To reduce user interaction time, one slice of the 3D CT image is manually delineated as prior model and then other contours are automatically and recursively delineated by using deformable registration. As shown in Figure 1(b), the previous slice is set as template image, and the next slice is set as target image. The first two steps, feature points detection and association, are conducted by Speeded-Up Robust Features (SURF) [12]. These points contain the local features and also keep invariant to the environment variation. Then, Thin-Plate Spline (TPS) [13] is employed to generate the deformation vector field (DVF) based on the displacement vectors of associated feature points. In the fourth step, the contour transformation is obtained from the DVF. Finally, Snake is introduced as refinement and modification approach to drive the initial contours towards the desired segmented object in the image.

### 2.2. Tissue Feature Detection and Association

Detection and association of distinct tissue feature points on two images play an important role in contour propagation [14]. SURF detector is the accelerated version of the classical Scale-Invariant Feature Transform (SIFT) [15], with the same matching quality. This is considered a beneficial approach because of its distinctive invariant features and robustness to affine distortion, noise, and intensity changes.

**Figure 2:**
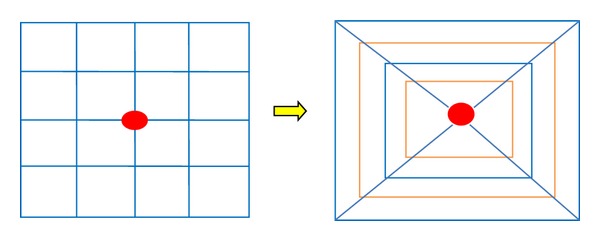
The SURF descriptor (left) and GDLOH descriptor (right). The GDLOH regards a concentric rectangle grid different radius and 4 in angular direction.

With regard to the feature descriptor, an improved version of SURF, gradient distance-location-orientation histogram (GDLOH) [16], is considered to be more distinctive. GDLOH descriptor, as shown in Figure 2, contains 16 square subregions with four principal characters: the frame center coordination (*x*, *y*), the scale *σ*, and the orientation *θ*. For each square, the Haar wavelet responses in horizontal direction and vertical direction are computed and summed. In this way, the histogram of 16 frames with 4 dimension gradient vector around the center can obtain 64 dimensional feature descriptors.

The descriptor is employed to search the best association candidate for each feature point by identifying its nearest neighbor in the database of points from testing images. The nearest neighbor is defined as points' minimum Euclidean distance. In this method, interrelationship among the descriptor's elements is also considered in the distance calculation. The expression can be defined as follows:

(1) $$\begin{array}{c} D\left(P,Q\right)=\left(\sum P^{2}\left[i\right]+\sum Q^{2}\left[i\right]\right)*\frac{\sum {\left(P\left[i\right]-Q\left[i\right]\right)}^{2}}{\left(2*\sum P\left[i\right]*Q\left[i\right]\right)}. \end{array}$$

In Cartesian coordinates, *P* = (*p*_1_, *p*_2_,…, *p*_*n*_) is the descriptor of the feature point *P*(*x*, *y*), and *Q* = (*q*_1_, *q*_2_,…, *q*_*n*_) is the descriptor of the feature point *Q*(*x*, *y*). Furthermore, the coordinates of the points *P*, *Q* can be confined in 10∗10 pixels according to the image rotation.

### 2.3. TPS Transformation

Thin-Plate Splines (TPS) were introduced in geometric designs by Duchon [17]. This has been widely used as deformable transformation model in image registration. In the proposed method, TPS transformation warps the template image to match the target image pixel-by-pixel. Mathematically, this consists in an optimization problem, in which a set of transformation parameters transform the pixels in the template image to their corresponding pixels in the target image.

To find the transformation matrix a TPS deformable model *T*(*X*) was employed to map an arbitrary pixel from the template image to that on the target image [18]. The function was defined as

(2) $$\begin{array}{c} f\left(u^{\prime },v^{\prime }\right)=a_{1}+a_{u}u+a_{v}v+\sum_{i=0}^{n-1}w_{i}U\left(\left|p_{i}-\left(u,v\right)\right|\right), \end{array}$$

where  *P*_*i*_  are control points coordinates in the template image and *U* is a basis function for measuring the distance. *W* = (*w*_1_, *w*_2_,…, *w*_*n*_) and *a*_1_, *a*_*u*_, *a*_*v*_ stand for the weighting vector and the coefficients, which were computed from series of matrices. These matrices were constructed using a pair of matched control points in the template image (*x*_*i*_, *y*_*i*_) and the target image (*u*_*i*_, *v*_*i*_). Major steps are presented as follows.

(1) *P*_1_ = (*x*_1_, *y*_1_), *P*_2_ = (*x*_2_, *y*_2_),…, *P*_*n*_ = (*x*_*n*_, *y*_*n*_) are *n* control points in the template image; the distance between point *i* and *j* is defined as *r*_*ij*_ = |*P*_*i*_ − *P*_*j*_|. Consider the following:
  (3) $$\begin{array}{c} P=\left[\begin{array}{ccc} 1 & x_{1} & y_{1}\\ 1 & x_{2} & y_{2}\\ \vdots & \vdots & \vdots \\ 1 & x_{n} & y_{n} \end{array}\right],\\ K=\left[\begin{array}{cccc} 0 & U\left(r_{12}\right) & \cdots & U\left(r_{1n}\right)\\ U\left(r_{21}\right) & 0 & \cdots & U\left(r_{2n}\right)\\ \vdots & \vdots & \vdots & \vdots \\ U\left(r_{n1}\right) & U\left(r_{n2}\right) & \cdots & 0 \end{array}\right],\\ L=\left[\begin{array}{cc} K & P\\ P^{T} & 0 \end{array}\right], \end{array}$$
 where *O* represents 4∗4 matrix of zeros and *U* is a basic function $U\left(r\right)=\sqrt{r^{2}\log r^{2}}$.
(2) *Q*_1_ = (*u*_1_, *v*_1_), *Q*_2_ = (*u*_2_, *v*_2_),…, *Q*_*n*_ = (*u*_*n*_, *v*_*n*_) represent *n* corresponding control points in target image. Construct matrices
  (4) $$\begin{array}{c} V=\left[\begin{array}{cccc} u_{1} & u_{2} & \cdots & u_{n}\\ v_{1} & v_{2} & \cdots & v_{n} \end{array}\right],\\ Y={\left(\begin{array}{cccc} V\;|\; & 0 & 0 & 0 \end{array}\right)}^{T}. \end{array}$$
 The weighting vector *W* = (*w*_1_, *w*_2_,…, *w*_*n*_) and the coefficients *a*_1_, *a*_*u*_, *a*_*v*_ can be computed by the equation
  (5) $$\begin{array}{c} L^{-1}Y={\left(\begin{array}{cccc} W\;|\; & a_{1} & a_{u} & a_{v} \end{array}\right)}^{T}. \end{array}$$
(3) The elements of *L* ^−1^ *Y* can be used to define a function *f*(*u*, *v*).

### 2.4. Active Contour (Snake)

The above contour propagation was based on the manual delineation and deformable registration; this could cause some potential artifacts or distortion. Adapted active contour was proposed to solve this issue. This model is an energy-minimizing spline guided by internal and external forces, which are responsible for driving the contour to the desired local minimum or pulling it towards features such as lines or edges. Its energy function can be represented as

(6) Esnake∗=∫01(α∗Econt(v(s))+β∗Ecurv(v(s))     +γ∗Eimage(v(s)))ds,

where *v*(*s*) = (*x*(*s*), *y*(*s*)) stands for the contour parameter, *E*_cont_ stands for the continuity energy, *E*_curv_ stands for the internal energy of the spline due to bend and smooth, *E*_image_ stands for the external image forces, and the three parameters *α*, *β*, and *γ* stand for the weight coefficient of *E*_cont_, *E*_curv_, and *E*_image_, and they all range from 0 to 1. The optimization of the parameters has been conducted by experiments with 3 parameters *α*, *β*, and *γ* equal to 0.15 ± 0.08, 0.2 ± 0.08, and 0.85 ± 0.06, respectively. Moreover, a band was employed to limit the range of contour motion. According to the experiment results, a band width of 3 to 9 pixels has been proposed for 512∗512 lung CT image.

### 2.5. Case Study and Evaluation

The CT images were acquired with a GE Discovery-ST CT scanner (GE Medical System, Milwaukee, WI, USA). The proposed method was developed using the Insight Segmentation and Registration Toolkit (ITK) [19] and Open Source Computer Vision (OpenCV) [20]. ITK is an open-source and cross-platform image processing software developed by the National Library of Medicine. OpenCV is a library of programming functions developed by Intel. In addition, VOLVIEW [21], PARAVIEW [22], and The Visualization ToolKit (VTK) [23] have been used for image visualization. The image sets for all the patients were reconstructed with a 2.5 mm slice thickness. Each CT slice was discretized into 512∗512 pixels. About 400 slices and nearly 1000 contours were tested in the evaluation.

To quantitatively evaluate the accuracy of our method, the Jaccard similarity (JS) [24, 25] between the automatic and manual segmentation was calculated as follows:

(7) $$\begin{array}{c} \text{JS}=\left(\frac{S_{\text{auto}}⋂S_{\text{manual}}}{S_{\text{auto}}⋃S_{\text{manual}}}\right), \end{array}$$

where *S*_auto_ is the area of the autosegmentation and *S*_manual_ is the area of the manual segmentation. The value of JS is defined from 0 to 1, where 0 indicates no overlapped regions and 1 indicates that these two regions are the perfect overlap. Area measures, such as JS, can give a good estimate of expert agreement; however, they are much insensitive to boundary errors in the segmentation. To provide additional information, Hausdorff distance (HD) [26] between autodelineation and the manual delineation is given for estimating mismatch degree. HD measures the maximum and minimum distance between two contour sets, and it can be used as metric of similarity between two contours superimposed together.

## 3. Results

**Figure 3:**
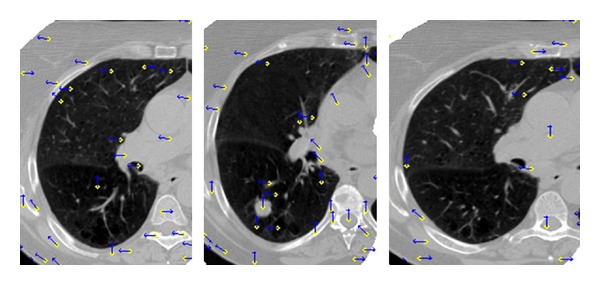
Displacement vectors of control points generated by the TPS transformation in three axial images. The yellow points stand for the control points, and the blue arrows stand for the displacement direction.

Figure 3 shows the displacement vectors of the control points generated by the TPS transformation; the three images show three different lung slices. The yellow points represent the control points, and the blue arrows stand for the displacement direction. At least 30 control points were obtained in each slice; this has satisfied the requirement of the TPS transformation [27].

**Figure 4:**
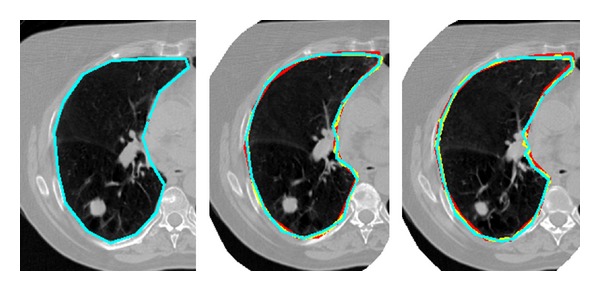
Process of the contour propagation and refinement, from the manual delineation to the final automatic contour. Three images stand for three sets of sequential slices. The cyan lines stand for the manual delineation, the red lines stand for the initial contour by SURF-TPS registration, and the yellow lines stand for the final contour refined by Snake. Yellow contours match the cyan better.

Normal tissue must be considered in radiotherapy plan, whose radiation sensitivity influences the prescribed radiation. In case of lung cancer, the organs at risk (OAR) mainly include lungs and spinal cord [28].  Figure 4 shows the process of the contour propagation and refinement, starting from the initial manual delineation to the final automatic contour. The three images represent a set of sequential slices. The cyan lines represent the manual delineation, the red lines represent the initial contour by SURF-TPS registration, and the yellow lines represent the final contour refined by Snake. It is clear that the yellow lines have much better consistency with the cyan lines as compared with the red lines, especially near the pleura. Since distribution of the pulmonary artery and bronchia is cluttered, distinctive local features are not easily discriminated by the fully automatic approach. Furthermore, after the initial propagation, the JS between the reds and cyans has reached 0.93 ± 0.08.

**Figure 5:**
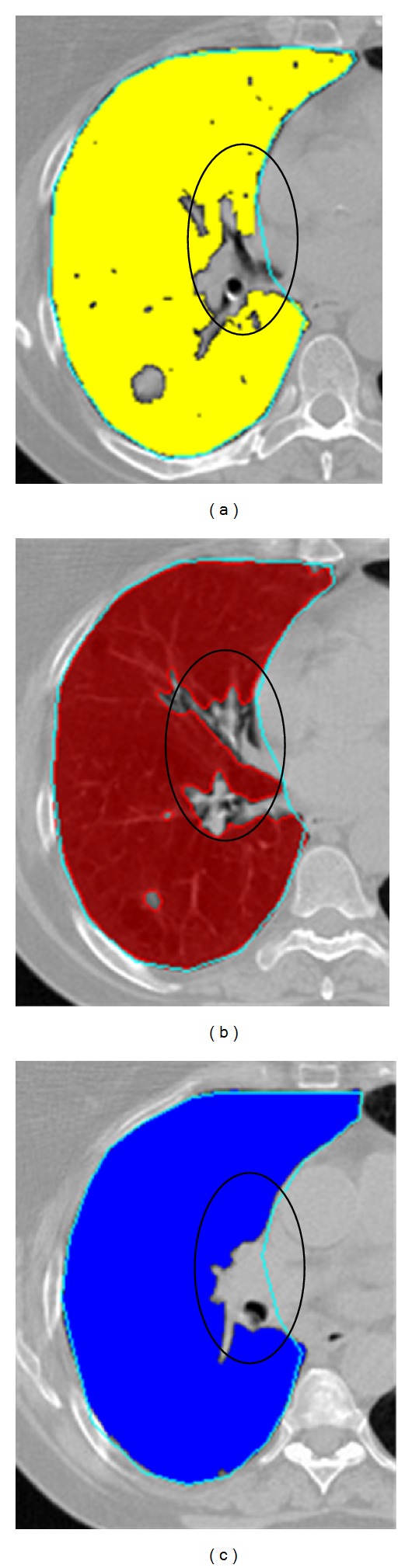
Comparison between fully automatic segmented methods and the manual delineation. The cyan contours were manual delineated contours. (a) Flood fill algorithm (yellow); (b) active contour (red); (c) watershed algorithm (blue). The region embraced by the black ellipse failed to be delineated by these automatic segmentations.

Figure 5 shows three lung segmented results conducted by fully automatic methods compared with the manual standard. Here, three classical algorithms have been chosen: watershed, flood fill, and active contour. The active contour used in the experiment, which has added the narrow band constraint proposed by Mille [29], is one of the popular extensions of the original Snake. The cyan contour represents the manual standard delineated manually, the yellow region in (a) is generated by flood fill algorithm, the red region in (b) is by active contour, and the blue region in (c) is by watershed algorithm. Notice that all three regions failed to embrace the hilus pulmonis near the mediastinum, indicated by the black ellipse, while our proposed method can reach the requirement (as shown in Figure 4). In addition, as these automatic algorithms highly depend on the initial seeds to start the driving, the selection of the seeds should be much more careful. In this way, it would raise potential delineation time and risk. The JS between the segmented region and the cyan surrounded one can only reach 0.85 ± 0.06; this value is much lower than the one found with the proposed method. In the experiment, since active contour and watershed algorithms, which highly rely on the intensity and seed information, do not perform well in the spinal cord and tumor delineation, we will mainly introduce the flood fill algorithm in the following to compare it with the results of our proposed method.

**Figure 6:**
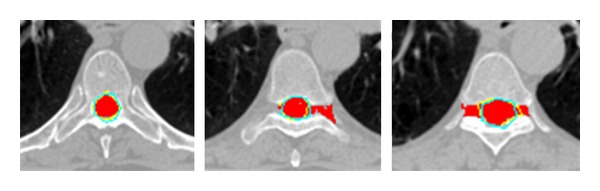
Three cases of delineation results conducted by our proposed method and the flood fill algorithm, respectively. The cyan contour represents the manual delineation, the yellow is automatically delineated by our proposed method, and the red region is generated by flood fill algorithm.

With respect to the spinal cord, which is more sensitive to the radiation injury, more precise delineation was needed.  Figure 6 shows three cases of delineation results obtained from the proposed method and the flood fill method, respectively. As shown in Figure 5, better results were achieved by using flood fill method and then active contour method and watershed method. In Figure 6, the red region is generated by the flood fill method, the yellow contour is delineated by our proposed method, and the cyan contour stands for the manually delineated contour. It has been found that the yellow contours can better fit the cyan contours as compared to the red contours. In addition, as the intensity information is distributed differently from slice to slice, the parameter settings of the flood fill algorithm should be changed. For example, the optimal local scale parameter for lung is 12 ± 2, but for spinal cord it is reduced to 5 ± 1, which results in some uncertainty. The JS between the red region and the cyan surrounded region is about 0.65 ± 0.08, which is much lower than what the yellow contains.

**Figure 7:**
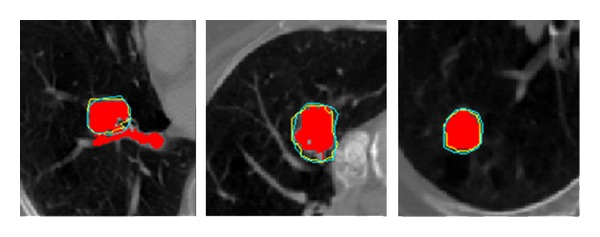
Three cases of lung tumor segmentation conducted by our proposed method and the flood fill algorithm, respectively. The tumors are located on the bronchia, hilus pulmonis, and lobe, respectively. The cyan contour represents the manual delineation, the yellow contour is generated by our proposed method, and the red region is generated by flood fill algorithm.

Figure 7 shows three cases of lung tumor segmentation obtained from our proposed method and the flood fill algorithm, respectively. The cyan contour represents the manual delineation, the yellow contour is generated by our proposed method, and the red region is generated by flood fill algorithm. These three tumors are located on the bronchia, hilus pulmonis, and lobe, respectively. As there is no obvious intensity difference among the tumor, bronchia, and hilus, the red contour tends to be more inconsistent with the cyan contour, while the yellow contour that can better fit the cyan contour well overcomes this problem. The JS between the yellows and the cyan is nearly 0.88 ± 0.5, which is much higher than the JS between the reds and the cyan.

Since the contour delineation of tumor and organ at risk (OAR) is very important for treatment planning, the feasibility and accuracy of the proposed method should be reliably tested. There are five lung cancer patients with over 400 CT slices for the verification. The position and size of the tumors differ from patient to patient.  Table 1 shows the JS by using the proposed method and the flood fill method. For patients 1 and 3, tumors are located on the lung lobe with much more apparent local features, while for the other three patients, tumors are located near the pleura, bronchia, and hilus pulmonis. Features are not apparent. It is easy to find that the JS of the first and the third patients, obtained from flood fill method, is much higher than that of the second, fourth, and fifth. On the contrary, the JS obtained from the proposed method presents small changes for all patients. When the delineation goes to the spinal cord with efferent nerves, the contour driven by flood fill tends to be less robust, and the JS between it and the manual standard becomes lower. The percentage gains of the JS between these two methods are also listed. The gains for lung and spinal cord remain steady around 0.10, but for tumor, it changes greatly among different cases. The maximum JS gain for lung, spine cord, and tumor has reached values around 12%, 17%, and 24%, respectively.

Table 2 shows the statistical value of mean and maximum of HD for lung, spinal cord, and tumor, respectively. As the tumor position and size differ from patient to patient, the HD should be listed respectively. It is clear that the HD improvement in lung is the biggest, reaching a value around 90%. The HD improvement in spinal cord can reach nearly 40%. On the contrary, for tumor, the HD improvements have a different representation. Its maximum HD improvement has, respectively, reached 90%, 45%, and 72%, according to the different tumor location. These results can be considered as a great progress in segmentation with no distinctive local feature.

## 4. Discussions

In this work, a feature-based recursive deformable registration strategy was proposed. In routine clinical procedure, target volume and OAR for lung cancer are manually delineated. Therefore, reliable automatic contour delineation may have a substantial impact on treatment planning. The proposed method takes a manual delineated slice as prior knowledge to recursively propagate the contour slice by slice. Here, the middle slice of the whole lung has been chosen as an initial manual delineated slice; this choice doubles the propagation distance. The total 2D delineation time can be dramatically reduced compared with the conventional 3D contour segmentation. Since the initial propagated contour's quality largely depends on the similarity between two adjacent slices, slice thickness turns to be a critical factor for autodelineation.

Feature-based deformable registration [30] is used to propagate the contour since it contains both local and global feature information and can overcome the low resolution of CT image. SURF is a popular local feature detection method, which has a good robustness to variation of the scale, luminance, rotation, and blurring. After registration, an adapted Snake is employed to refine and modify the rough outline. The proposed method outperforms other fully automatic segmentation algorithms, such as the classical water-based and extensional active contour approach, as shown in Figure 5. In the picture, it also can be seen that the hilus pulmonis near the mediastinum was surrounded by our autopropagated contour as shown in Figure 4.

However, the proposed method still presents some weakness. For example, it much relies on the slice thickness. In fact, if the thickness is set excessively large, the quality of registration would decline, directly influencing the autodelineation. According to experiment measurements, 3 mm is suggested as the maximum thickness for achieving good performance. The adapted Snake should be further optimized, since the deformation still causes some instabilities. In addition, as the influence of prior knowledge would decrease with the propagation distance, the length of the propagation routine becomes more and more limited. For lung delineation, the maximum propagation distance with 20 slices has given optimal results. All in all, the delineation time was reduced drastically, while the accuracy remains high.

## 5. Conclusion

Automatic accurate target delineation plays an important role in radiotherapy allowing escalating tumor doses without increasing the toxicity of critical normal structures, especially in pulmonary treatment planning. The proposed method provides a novel approach combining the delineation experience from the physicists with high speed and reliability from the automatic algorithm. The advantages of the proposed method are as follows. Firstly, it largely reduces the delineation time by automatic contour propagation compared to manual delineation slice by slice. Secondly, the prior knowledge is well preserved by SURF-TPS registration increasing the accuracy of contour propagation. Thirdly, the refinement and modification by adapted Snake are beneficial for further precision upgrade. The proposed method will find practical and useful application in clinical treatment planning in radiotherapy.

## Figures and Tables

**Table 1:** JS comparison of five lung cancer patients by using the proposed method and the flood fill algorithm.

Patient	Target	The JS		Percentage gains
		Proposed method	Flood fill method	
Patient 1	Lung	0.9458	0.8540	9.71%
	Spinal cord	0.8520	0.7018	17.63%
	Tumor	0.9447	0.9334	1.20%

Patient 2	Lung	0.9544	0.8658	9.28%
	Spinal cord	0.8402	0.6875	18.17%
	Tumor	0.8589	0.7569	11.88%

Patient 3	Lung	0.9541	0.8320	12.80%
	Spinal cord	0.8654	0.7468	13.70%
	Tumor	0.9014	0.8654	3.99%

Patient 4	Lung	0.9440	0.8214	12.99%
	Spinal cord	0.8598	0.7025	18.29%
	Tumor	0.8958	0.6475	27.72%

Patient 5	Lung	0.9486	0.8258	12.95%
	Spinal cord	0.8475	0.6732	20.57%
	Tumor	0.8858	0.7854	11.33%

**Table 2:** HD comparison between the proposed method and the Flood fill method.

Target	Lung	Spinal cord	Tumor				
			1	2	3	4	5
HD							
Flood fill	54.21	6.07	3.23	9.18	2.23	20.42	6.18
Proposed	5.04	3.45	3.35	2.53	2.15	5.84	4.85
Improvement (%)	90.70	43.16	−3.72	72.44	3.59	71.40	21.52

Mean HD							
Flood fill	7.58	1.71	0.63	2.85	0.83	3.38	2.61
Proposed	0.91	0.92	0.59	1.09	0.68	1.67	1.83
Improvement (%)	87.99	46.20	6.35	61.75	18.07	50.59	29.89
